# Introducing human 3D skin models as a new serological diagnostic tool for severe autoimmune bullous diseases

**DOI:** 10.3389/fimmu.2025.1661851

**Published:** 2025-09-25

**Authors:** Laura Huth, Ruth Heise, Yvonne Marquardt, Manuela Jansen, David Kluwig, Morna Friederike Schmidt, Nicole Albuscheit, Verena von Felbert, CTC-A Translation Group, Stefan Jockenhoevel, Sebastian Huth, Amir S. Yazdi, Jens Malte Baron

**Affiliations:** ^1^ Department of Dermatology and Allergology, Uniklinik RWTH Aachen, Aachen, Germany; ^2^ DWI Leibniz-Institute for Interactive Materials, Aachen, Germany; ^3^ CTC-A – Center for Translational & Clinical Research, Uniklinik RWTH Aachen, Aachen, Germany

**Keywords:** human 3D skin models, autoimmune bullous diseases, pemphigus vulgaris, bullous pemphigoid, indirect immunofluorescence, serological diagnosis

## Abstract

Autoimmune bullous diseases (AIBDs) are acquired disorders characterized by autoantibodies targeting structural proteins of the skin and mucous membranes, resulting in blister formation. In pemphigus, pathogenic autoantibodies primarily directed against desmosomal adhesion proteins (desmoglein 1 and 3), disrupt epidermal cell-cell adhesion, leading to intraepidermal blister formation. In contrast, pemphigoid diseases are marked by subepidermal blistering due to autoantibodies against hemidesmosomal proteins, such as BP180 and BP230, located in the basement membrane zone. Diagnosis of AIBDs is based on clinical presentation, histolopathology, direct immunofluorescence, and serological analyses. Specific circulating autoantibodies can be identified using indirect immunofluorescence (IIF), which conventionally relies on animal-derived tissues, such as monkey esophagus, as substrates. This study aimed to develop a standardized *in vitro* diagnostic platform that eliminates the need for animal tissues. Human 3D skin models composed of dermal fibroblasts and epidermal keratinocytes were generated. Cryosections from these models were evaluated by IIF using sera from 34 patients diagnosed with either pemphigus vulgaris, pemphigus foliaceus, or bullous pemphigoid. As expected, sera from patients with pemphigus diseases produced the characteristic intercellular fluorescence pattern within the epidermis, while sera from pemphigoid patients exhibited staining along the basement membrane zone. These staining patterns precisely matched those obtained using monkey esophagus tissue. Notably, the 3D skin model demonstrated a significantly higher diagnostic sensitivity compared to the conventional monkey esophagus substrate. In summary, cryosections from human 3D skin models provide a sensitive and animal-free alternative for the serological diagnosis of AIBDs, accurately reproducing disease-specific immunofluorescence pattern.

## 1 Introduction

Autoimmune bullous diseases (AIBDs) are a heterogeneous group of autoantibody-mediated disorders that manifest with blisters or erosions on the skin and/or mucous membranes (1, 2). Two major categories of AIBDs are pemphigus diseases and autoimmune bullous diseases of the pemphigoid type (3). Pemphigus diseases can be classified in different forms: pemphigus vulgaris (PV, 78-80%), pemphigus foliaceus (PF, about 20%), paraneoplastic pemphigus (PNP, about 5%), and IgA pemphigus (1-3%) (2). All these forms are characterized by the production of autoantibodies directed against desmosomal proteins, leading to acantholysis and intraepidermal blister formation in the skin and/or mucous membranes (3, 4). PF is typically associated with anti-Dsg1 antibodies alone, while PV patients may exhibit either anti-Dsg3 antibodies (mucosal-dominant type) or both anti-Dsg3 and anti-Dsg1 antibodies (mucocutaneous type) (3). However, a group of Pemphigus patients only carry antibodies against desmoplakins, desmocollins or envoplakin, which are currently not detectable in commercially available ELISA kits (5).The main disorders of autoimmune bullous diseases of the pemphigoid type include bullous pemphigoid (BP), mucous membrane pemphigoid (MMP), and epidermolysis bullosa acquisita (EBA) (4). These conditions are marked by autoantibodies against hemidesmosomal proteins, particularly BP230 and BP180, resulting in subepidermal blister formation (6).

The prevalence of AIBDs varies significantly across geographic regions and ethnic groups (4). PV is the most common pemphigus form globally, with European incidence rates ranging from 0.5 to 8 per million per year (4). BP, the most frequent AIBD in Germany, shows incidence rates ranging from 2.5 to 42.8 per million annually across Europe and predominantly affects individuals over the age of 70 (1, 4).

If left untreated, AIBDs can be potentially life-threatening due to complications such as superinfections, loss of fluid, and severely restricted food intake (7). Accurate diagnosis of AIBDs requires more than clinical evaluation alone. Therefore, a comprehensive, multi-step diagnostic process is standard, including dermatological assessment, histopathology features, direct (DIF) and indirect immunofluorescence (IIF), and enzyme-linked immunosorbent assay (ELISA) (8). DIF is considered the gold standard for detecting tissue-bound antibodies in perilesional skin biopsies while circulating antibodies can be detected via both ELISA and IIF. ELISA allows a quantitative analysis of autoantibodies, whereas IIF employs animals tissue (monkey esophagus or rat bladder) as substrate to detect antibodies (1, 3, 9). In this context, reconstructed human skin equivalents represent an alternative to animal testing, as they comply with the 3R principle (Replacement, Reduction, and Refinement) and offer a way to meet the requirements of regulatory authorities (10, 11). Artificially reconstructed skin equivalents consist of dermal and epidermal layers and therefore closely resemble natural skin (12). They offer standardized platforms for toxicity testing, safety assessments, and basic research into skin biology, wound healing, and skin disease pathogenesis (10). Currently, no 3R-compliant alternatives are commercially available on the market. Based on this need, the aim of this study was to develop a standardized *in vitro* diagnostic tool that replaces animal-derived tissues in accordance with the 3R principle.

## 2 Materials and methods

### 2.1 Patient samples and study design

Sera from 34 patients with pemphigus vulgaris (3), pemphigus foliaceus (5) and bullous pemphigoid (26) were collected. The patients were between 41 and 94 years old, of which 18 were female and 16 male. As part of routine diagnostics, DIF, ELISA and IIF on monkey esophagus were performed and the results were recorded for the respective patients. In addition, IIF was performed on cryosections of a human full skin 3D skin model incubated with patient sera. These results were compared with those of routine diagnostics. Essential technical documentation for the test device was prepared following the *In-Vitro*-Diagnostic Regulation (IVDR) and applying quality standards for medical devices. Starting from user needs, the intended purpose for the new tool was determined, requirements defined, and risks assessed, to assure safe and reproducible production and testing of the cryosections of the 3D skin models and to obtain reliable diagnostic results. This study was conducted according to the Declaration of Helsinki Principles and was approved by the ethical committee of the University Hospital, RWTH Aachen, Germany (EK 349-21). A written informed consent was obtained from all participants.

### 2.2 3D skin models

Normal human epidermal keratinocytes (NHEK; C-120006, PromoCell, Heidelberg, Germany) and normal human dermal fibroblast (NHDF; CC-2511, Lonza, Basel, Switzerland) were cultured according to the manufacturer’s recommendations. Collagen-based 3D skin equivalents were performed as previously described (13, 14). In brief, to construct the dermal part of the skin equivalent, collagen gels were prepared by mixing eight volumes of ice-cold bovine collagen I solution (Advanced Biomatrix, Carlsbad, CA, USA) with one volume of ×10 concentrated Hank’s balanced salt solution (ThermoFisher, Waltham, Massachusetts, USA). After neutralization with 1 mol/L NaOH, one volume of NHDF suspended in FCS was added. The final concentration of NHDF in this gel solution was 2 × 10^5^ cells/mL. Four milliliters of this gel solution were poured into each polycarbonate membrane insert (3.0 μm pore size; Corning, NY, USA) and placed in six-well plates. Following complete polymerisation, gels were covered with DMEM and incubated in a humidified atmosphere at 37 °C and 5% CO_2_. Following day, approximately 2 × 10^6^ NHEK cells were seeded on each dermal equivalent and incubated at 37 °C and 5% CO_2_ for one day. Afterwards, 3D skin models were lifted to the air–liquid interphase and cultivated for 14 days. A schematic overview is shown in **Supplementary Figure 1**. After 14 days the 3D skin models were harvested and embedded in Tissue-Tek^®^ O.C.T.™ compound (Sakura Finetek, Zoeterwoude, The Netherlands) for cryosectioning.

### 2.3 Enzyme-linked immunosorbent assay

All ELISA kits were purchased from Euroimmun (Lübeck, Germany) and performed according to the manufacturer’s instructions. ELISA kits for the determination of human autoantibodies against desmoglein 1 (EA 1495–4801 G) and desmoglein 3 (EA 1496–4801 G) from patient serum were used for the diagnosis of pemphigus vulgaris and foliaceus. In patients with bullous pemphigoid, ELISA assays against BP180 (EA 1502-4801–2 G) and BP230 (EA 1502-4801–1 G) were performed for diagnostic purposes. These results were obtained as part of the routine diagnostics in the Department for Dermatology and Allergology, Uniklinik RWTH Aachen.

### 2.4 Direct immunofluorescence

For DIF staining 4 µm sections were cut and incubated with fluorescein-conjugated antibodies to human IgG (1:10 dilution, Dako, Glostrup, Denmark), IgA (1:20 dilution, Dako), and C3 (1:10 dilution, Dako) for 30 min. Following immunostaining, the sections were washed with PBS and mounted with fluorescence mounting media (Dako) before examining with a fluorescence microscope (Leica, Wetzlar, Germany).

### 2.5 Indirect immunofluorescence

IIF on monkey esophagus was performed using the NOVA Lite^®^ Monkey Esophagus Kit (Werfen, Barcelona, Spain) according to the manufacturer’s instructions. In brief, monkey esophagus slides were incubated with patient serum (1:10) and appropriate controls [1:10; negative control serum, two positive control sera (one pemphigus, one pemphigoid)] for 30 min at room temperature. Afterwards unbound antibodies were washed off and then anti-human IgG fluorescein labeled conjugates were applied for 30 min at room temperature in the dark. After another washing step slides were viewed with a fluorescence microscope (Keyence Deutschland GmbH, Neu-Isenburg, Germany). IIF on human 3D skin models was performed exactly as just described, only the slides of monkey esophagus were replaced by 4 µm cryosections of the human 3D skin model.

## 3 Results

### 3.1 Control samples of AIBD stain human skin equivalents equally to monkey esophagus

**Figure 1:**
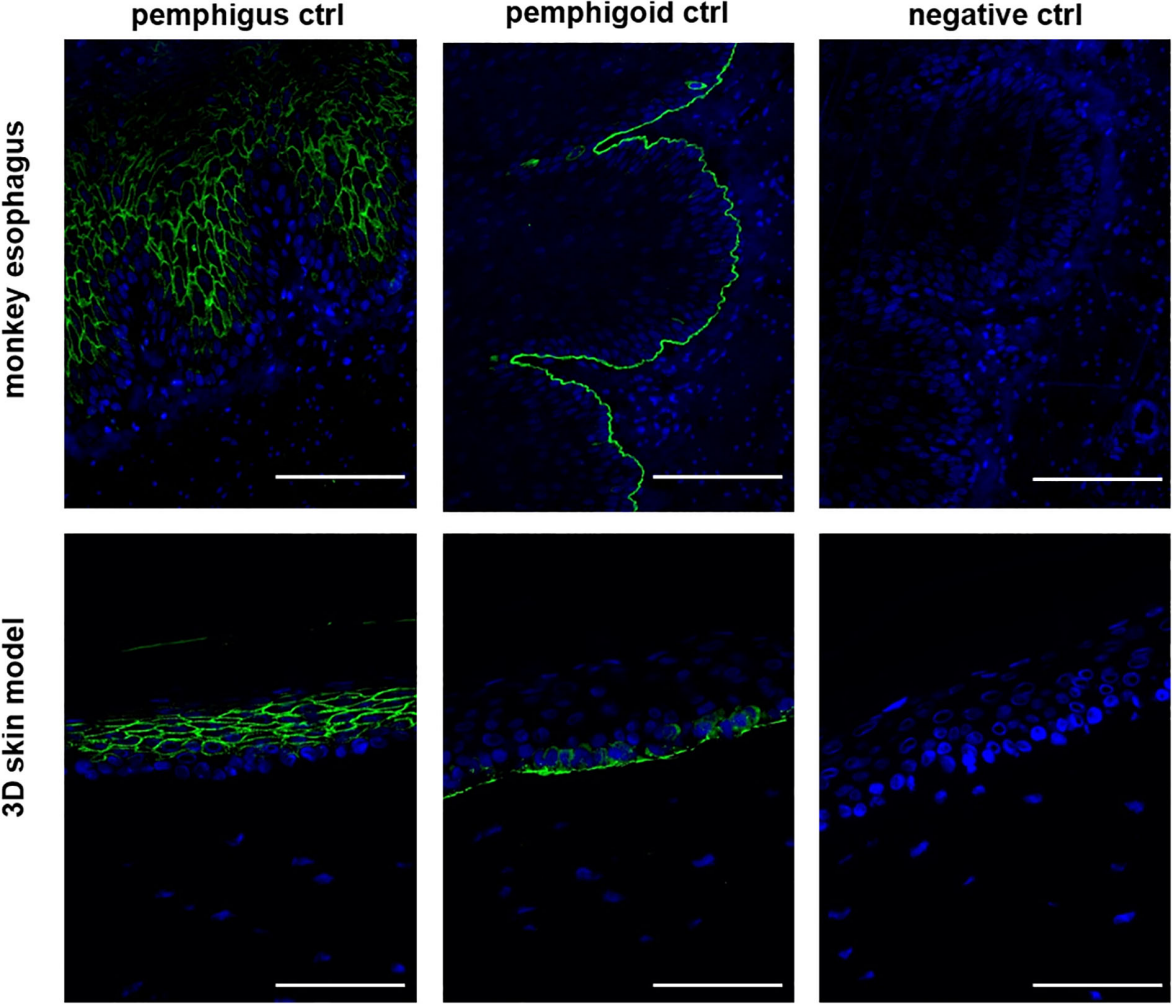
The 3D skin model is suitable for the diagnosis of AIBDs. In the IIF with the pemphigus and pemphigoid control, both the monkey esophagus and the 3D model as a substrate showed the same staining pattern. In the pemphigus control, intraepidermal fluorescence was detected, while the pemphigoid control exhibited staining along the basement membrane. The negative control showed no fluorescence and served as a control. All three controls are derived from human serum. Ctrl, control; magnification = 400x, scale bar = 100 µm.

As an initial step, we evaluated whether our human 3D skin model could reproduce the characteristic staining patterns required for the diagnosis of autoimmune blistering diseases (AIBDs), thereby assessing its diagnostic applicability. To this end, immunofluorescence staining was performed on tissue sections from the human 3D skin model using positive control sera for pemphigus and pemphigoid, and the results were compared to those obtained using monkey esophagus slides (**Figure 1**). The pemphigus control demonstrated the expected intercellular staining of the epidermis on both the monkey esophagus and the 3D skin model. Similarly, the pemphigoid control exhibited clear linear staining along the basement membrane in both substrates. No staining was observed in the negative control samples.

### 3.2 Human 3D skin models are superior to monkey esophagus in IIF

**Figure 2:**
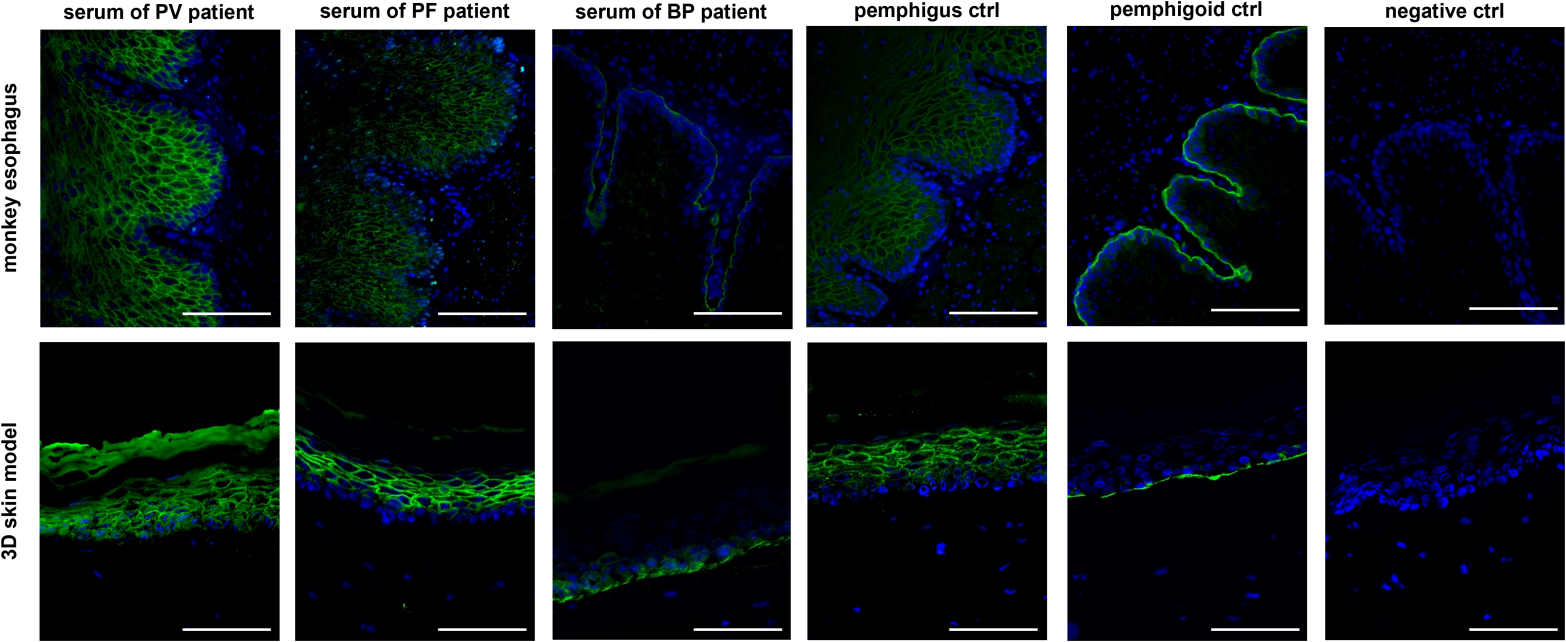
Comparison of IIF staining with patient sera of each disease group (PV, PF and BP) using monkey esophagus (upper row) as well as the 3D skin model (bottom row) as a substrate. The staining of the sera from the group of pemphigus diseases clearly showed a reticular intraepidermal staining, while in the IIF of patient sera with bullous pemphigoid a staining of the basement membrane could be detected. All three controls are derived from human serum and showed the expected color pattern. Representative images of all disease patterns are shown, whereby the sera of 34 patients were stained in total. Ctrl, control; magnification = 400x, scale bar = 100 µm.

**Figure 3:**
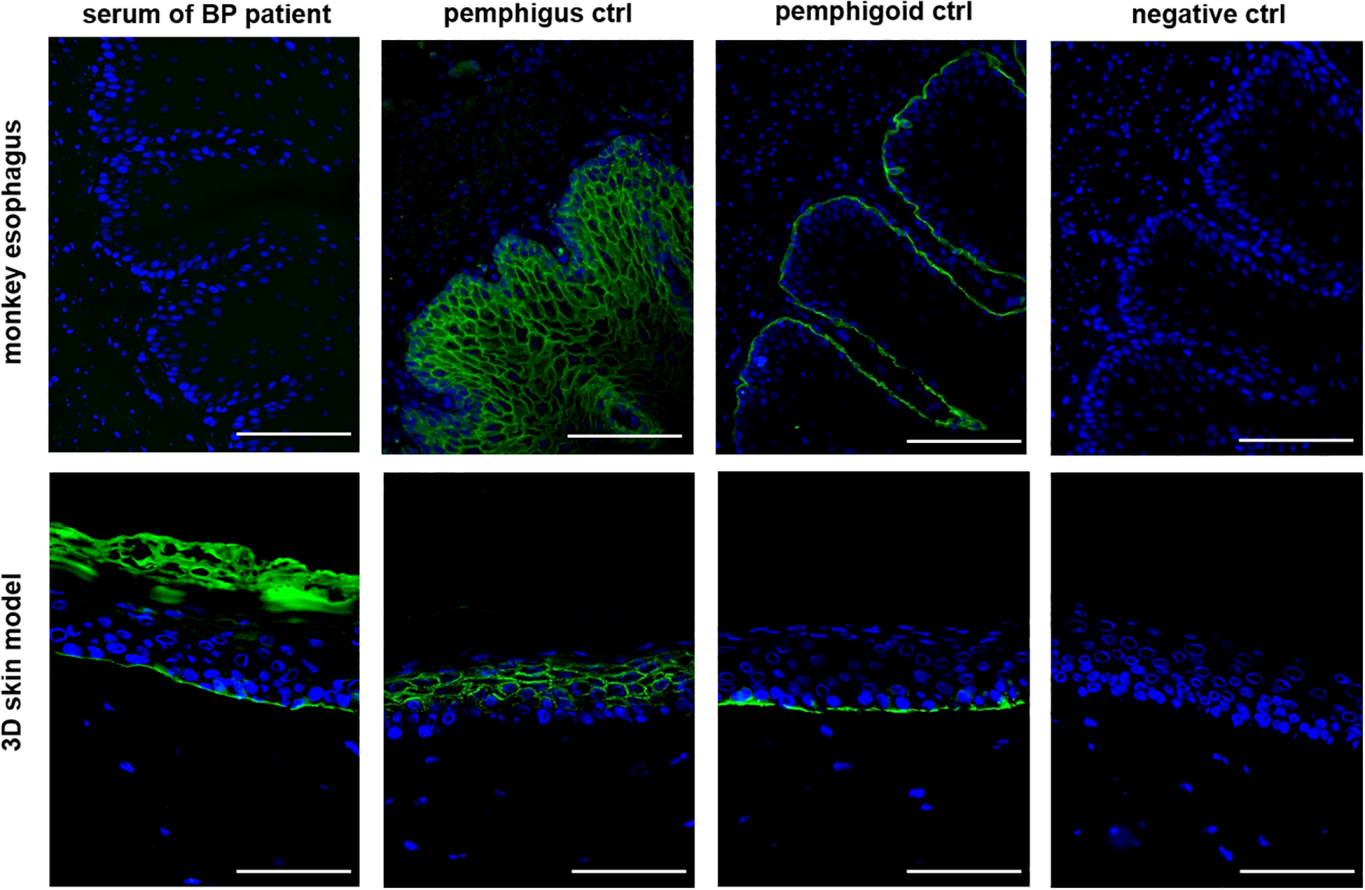
The 3D skin model as a substrate for IIF is more sensitive than the monkey esophagus. An example of a BP patient serum is shown in which no staining could be detected using the monkey esophagus, but clear staining of the basement membrane could be detected using the 3D skin model. All three controls are derived from human serum and showed the expected staining pattern. Ctrl = control, magnification = 400x, scale bar = 100 µm.

Subsequently, IIF was performed on all 34 patient sera using our human 3D skin model and compared to results obtained with the monkey esophagus substrate. Representative images from each disease group, namely PV, PF and BP, are shown in **Figure 2**. Sera from PV and PF patients exhibited the characteristic intercellular, net-like staining pattern within the epithelium, commonly referred to as the “chicken wire” or “honeycomb” pattern (1, 3, 15, 16). A similar smooth, reticular staining pattern was also observed in most epithelial layers of the pemphigus control sample. In contrast, sera from pemphigoid patients showed a linear staining pattern along the basement membrane zone, consistent with that observed in the pemphigoid control. These disease-specific staining patterns were consistently detected using both the monkey esophagus and the human 3D skin model as substrates. Notably, fluorescence localization in sections of the 3D skin model allowed differentiation between PV and PF sera, with the latter exhibiting a more intense signal in the superficial layers of the epidermis (**Supplementary Figure 2**). In the next step, all IIF results obtained with the 3D skin model were compared with diagnoses established through routine diagnostic methods (**Table 1**). Among the three pemphigus vulgaris patients, all routine diagnostic procedures (DIF, ELISA, IIF with monkey esophagus) as well as the new diagnostic test system (IIF using our 3D skin model) successfully established the diagnosis in 100% of cases (2/2 resp. 3/3; **Table 1**). For pemphigus foliaceus (PF) patients, the sensitivity of routine diagnostics varied: DIF detected 100% (3/3; **Table 1**), while ELISA and IIF with monkey esophagus detected 60% (3/5 each; **Table 1**). In contrast, IIF using the 3D skin model showed a higher sensitivity of 80% in PF patients (4/5; **Table 1**). In bullous pemphigoid (BP) patients, routine diagnostics detected 100% by DIF (23/23), 85% by ELISA (22/26), and 69% by IIF with monkey esophagus (18/26) (**Table 1**). The IIF using our 3D skin model identified all BP patients, achieving a sensitivity of 100% (26/26; **Table 1**). **Figure 3** illustrates an example of a BP patient diagnosed via the 3D skin model IIF but missed when using monkey esophagus as a substrate. A potential correlation between antibody titers determined by ELISA and fluorescence signal intensity of the IIF could not be detected (data not shown). Overall, this feasibility study showed that patients with AIBDs could be detected with varying degrees of sensitivity depending on the diagnostic method used. DIF achieved the highest sensitivity of 100% (28/28; **Table 1**). Our novel 3D skin model followed closely, with a sensitivity of 97% (33/34; **Table 1**). ELISA yielded a sensitivity of 82% (28/34; **Table 1**), while IIF using monkey esophagus substrate showed the lowest sensitivity at 71% (24/34; **Table 1**).

**Table 1:** Overview of the diagnoses of the study patients obtained using the various diagnostic procedures.

Patient	Routine diagnostics			New diagnostic tool
	DIF	ELISA	IIF (monkey esophagus)	IIF (3D skin model)
pemphigus vulgaris (PV)				
#1	n/a	+	+	+
#2	+	+	+	+
#3	+	+	+	+
pemphigus foliaceus (PF)				
#4	n/a	+	+	+
#5	n/a	+	–	+
#6	+	–	+	+
#7	+	+	+	+
#8	+	–	–	–
bullous pemphigoid (BP)				
#9	+	+	+	+
#10	+	+	–	+
#11	+	+	+	+
#12	+	+	–	+
#13	+	+	+	+
#14	+	+	+	+
#15	n/a	+	+	+
#16	+	+	+	+
#17	+	+	–	+
#18	+	+	+	+
#19	+	–	+	+
#20	+	+	+	+
#21	+	+	+	+
#22	+	–	–	+
#23	+	+	–	+
#24	+	+	+	+
#25	n/a	+	+	+
#26	+	–	–	+
#27	+	+	+	+
#28	+	–	–	+
#29	n/a	+	–	+
#30	+	+	+	+
#31	+	+	+	+
#32	+	+	+	+
#33	+	+	+	+
#34	+	+	+	+

+, positive result; -, negative result; n/a, not available.

## 4 Discussion

Diagnosis of autoimmune blistering diseases (AIBDs) is based on dermatological examination, histological analysis of skin lesion biopsies, direct immunofluorescence (DIF) using perilesional biopsies, and serum testing by enzyme-linked immunosorbent assay (ELISA), as well as indirect immunofluorescence (IIF) using monkey esophagus as substrate (17), with DIF being the gold standard in diagnosing AIBD. However, each of these diagnostic methods has limitations and must be combined to ensure accurate diagnosis and effective monitoring of treatment response (18) with both histology and DIF demand an invasive skin biopsy with the risks of a small operation. A further shortcoming of DIF is that it provides only limited information regarding the specific target antigens and cannot differentiate between various subtypes of pemphigoid diseases (1, 6, 19). Therefore, additional serological assays are often required to confirm and further characterize the diagnosis established by DIF (18). Conventionally, the serological diagnosis of AIBDs follows a multi-step approach, beginning with IIF screening, followed by antigen-specific assays such as ELISA (19) as only known, commercially available epitopes are detectable in ELISA.

Routine IIF testing is typically performed using cryosections of monkey esophagus, which serve as a substrate for detecting circulating antibodies directed against intraepidermal antigens (in pemphigus) or basement membrane zone components (in pemphigoid). However, a study by Emtenani et al. demonstrated that cryosections of normal human skin have a higher sensitivity for detecting BP180-NC16A-specific autoantibodies compared to monkey esophagus (17). In addition to these diagnostic limitations, ethical considerations have prompted growing interest in replacing animal-derived tissues with human-based or synthetic alternatives, in alignment with the 3Rs principle (Replacement, Reduction, and Refinement) (20).

The aim of our study was to develop a standardized *in vitro* diagnostic platform based on human tissue as a substitute for monkey esophagus in IIF assays. Human 3D skin models represent physiologically relevant *in vitro* systems that recapitulate all layers of human skin (dermis, basement membrane, and epidermis), closely mimicking native skin in terms of tissue architecture, gene expression, and metabolic activity (21). Our previous studies have demonstrated that these 3D skin models are reliable tools for investigating the pathophysiology of skin diseases (22–25), evaluating topical treatments and wound healing (26, 27), and exploring molecular effects of various laser systems (28–33).

In this study, we demonstrate that tissue sections of a standardized 3D skin model represents a true alternative to monkey esophagus as a substrate in the diagnosis of AIBDs. The sections of the 3D skin model consistently showed identical staining patterns to those observed with monkey esophagus, each characteristic of the corresponding pemphigus or pemphigoid disease group. However, it was demonstrated that sections of the 3D skin model are capable of reliably distinguishing between PV and PF based on distinct fluorescence patterns. Specifically, in PF, the fluorescence signal was markedly stronger in the superficial layers of the epidermis, corresponding to the regions where desmoglein 1 (DSG1) is predominantly expressed (8, 9). Moreover, in our study, we successfully compiled a cohort of 34 patients, enabling a direct comparison of the diagnostic sensitivity between our 3D skin model and established routine diagnostic methods. The application of our 3D skin model consistently produced reliable fluorescence signals. Across five independent batches, 34 patient sera were analyzed alongside appropriate controls, demonstrating a high degree of standardization and robustness.

In line with the literature, which considers direct immunofluorescence (DIF) the gold standard for diagnosing AIBDs with a reported sensitivity of up to 91% (19), we used DIF to make the definite diagnosis as in our study it demonstrated a sensitivity of 100%. The sensitivity of ELISA (82%) and IIF using monkey esophagus (71%) in our study was notably lower but consistent with previously published data (15, 34), thereby reinforcing the validity of our findings. Compared to these established methods, tissue sections from our 3D skin model achieved a sensitivity of 97%, demonstrating a clear diagnostic advantage over monkey esophagus. When used in combination with other diagnostic approaches, the 3D skin model significantly enhances the overall accuracy of AIBD diagnosis.

One limitation of our study is that we tested only sera from patients with confirmed AIBD. Including sera from healthy individuals as well as from patients with other dermatological diseases would have enabled us to assess potential cross-reactivity and false-positive results, i.e., the specificity of the 3D skin model sections. Follow-up studies are planned to both expand the AIBD serum cohort and incorporate additional negative controls. Another limitation of our study is the fact that the widely applied salt-split technique has not yet been successfully implemented in our 3D skin model. Nonetheless, we aim to establish this method in our 3D skin model in future studies.

In summary, we present that tissue sections from 3D human skin models can be used as a novel *in vitro* diagnostic tool for the detection of AIBDs, offering a viable replacement for the animal-derived substrates currently used in IIF. Such a substitution with our human 3D skin model would not only align with the principles of the 3Rs (Replacement, Reduction, and Refinement), but our clinical study also demonstrated that this human-derived material provides higher sensitivity in the detection of AIBDs.

## Data Availability

The raw data supporting the conclusions of this article will be made available by the authors, without undue reservation.

## Group members of CTC-A translation group

Sabine Küppers (https://orcid.org/0009-0007-6602-6309), Dorothea Baumann (https://orcid.org/0009-0009-6260-695X), Beate Tschöke (https://orcid.org/0009-0005-4853-6319), Rainer Schuckelt (https://orcid.org/0009-0004-3177-3169) and Myriam Lipprandt (https://orcid.org/0000-0001-9371-0551) on behalf of the CTC-A Translation Group.
