# Supplementary material for: Introducing human 3D skin models as a new serological diagnostic tool for severe autoimmune bullous diseases
Source: Front Immunol. 2025 Sep 25;16:1661851. doi: 10.3389/fimmu.2025.1661851 (PMC12507869; doi:10.3389/fimmu.2025.1661851)
Supplement: Supplementary file 1 [file Table1.docx]

Supplementary Material

# Supplementary Figures and Tables

## Supplementary Figures

**
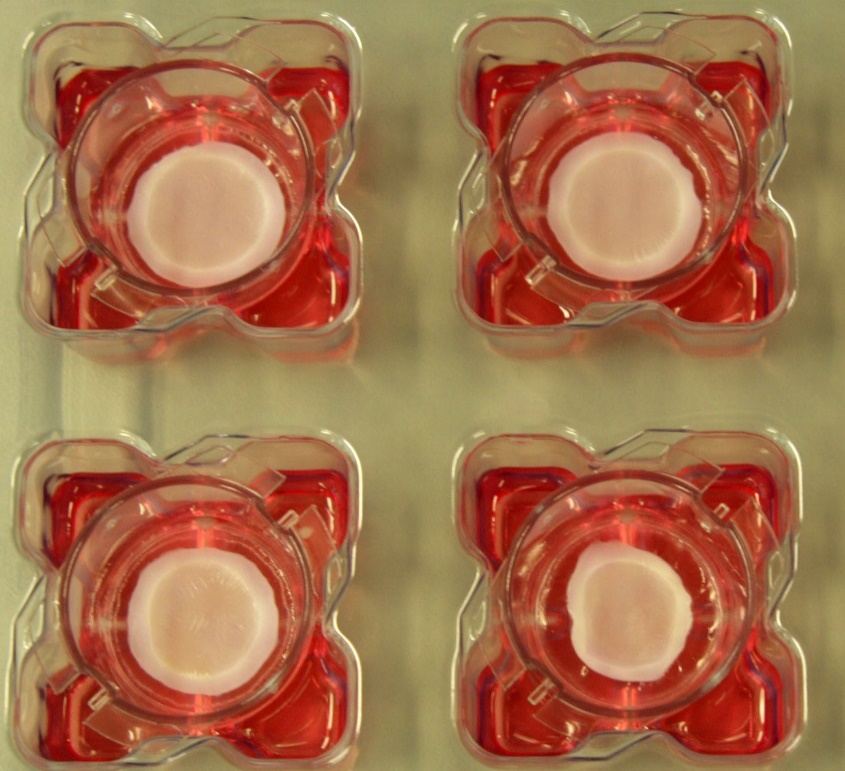
**
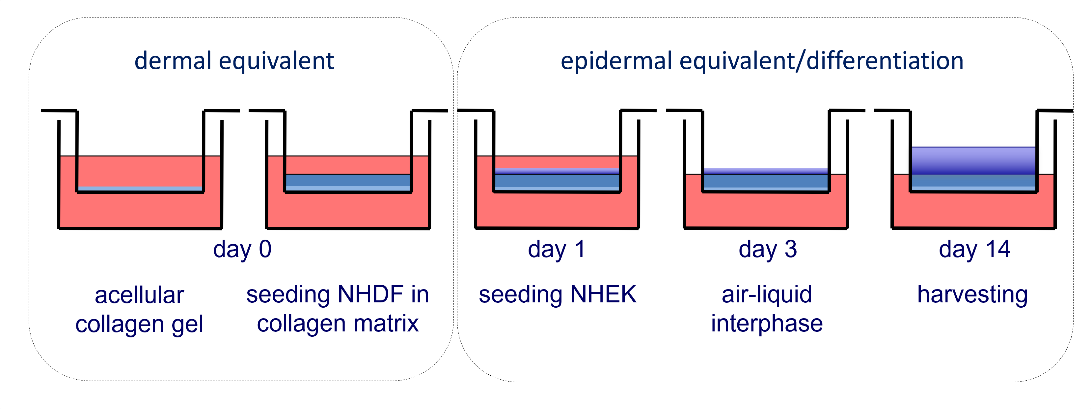


**Supplementary Figure 1.** Schematic overview of how the human 3D skin model was produced (left). Macroscopic picture of the fully differentiated 3D skin model (right).


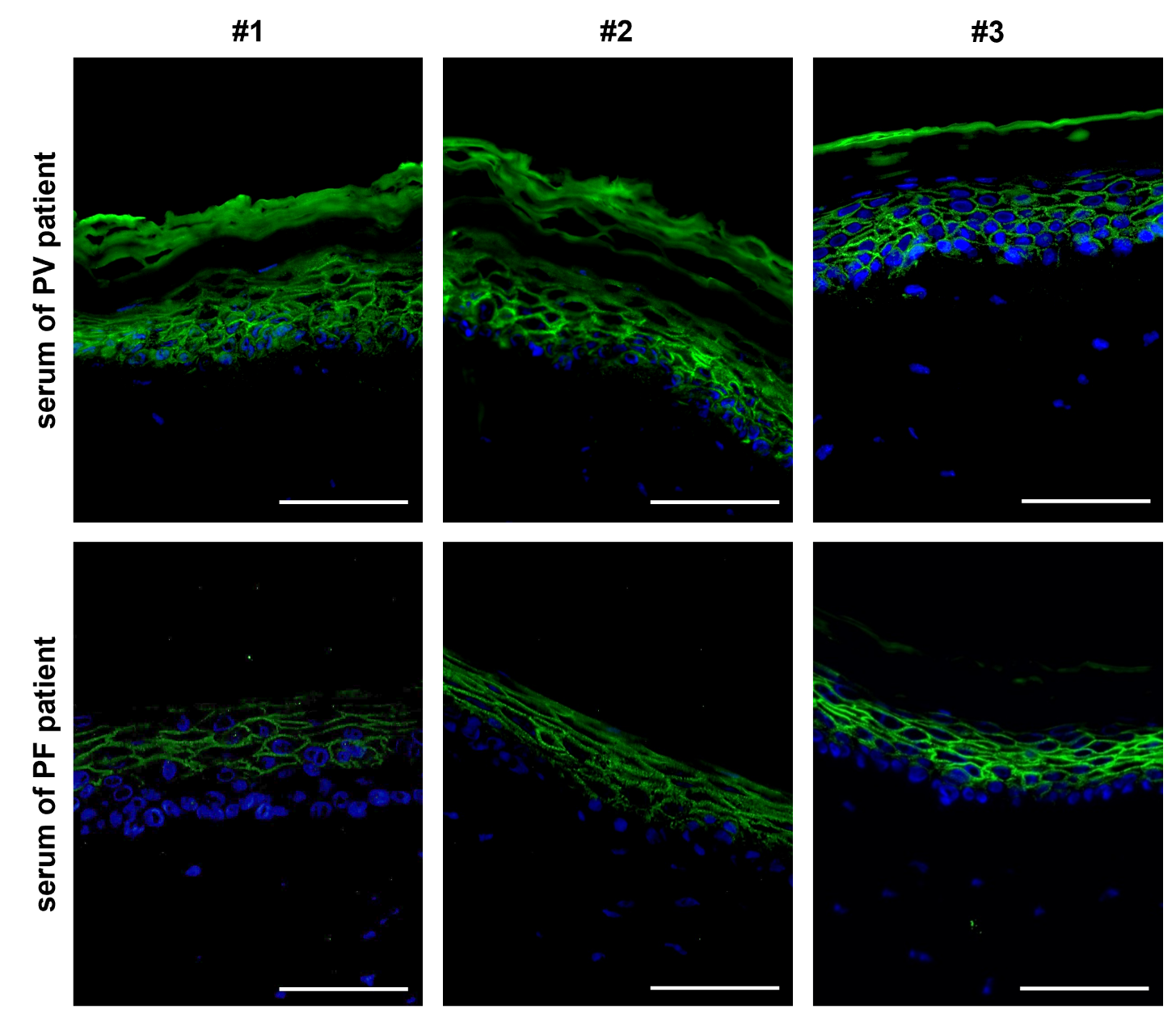


**Supplementary Figure 2.** Comparison of IIF staining with patient sera of 3 PV patients (upper row) and 3 PF patients (bottom row) using the 3D skin model as a substrate. The staining of the sera from the group of pemphigus vulgaris diseases clearly showed a reticular intraepidermal staining, while in the IIF of patient sera with pemphigus foliaceus a staining in the more superficial layers of the epidermis could be detected. Magnification = 400x, scale bar = 100 µm.
